# Smart Metering for Challenging Scenarios: A Low-Cost, Self-Powered and Non-Intrusive IoT Device †

**DOI:** 10.3390/s20247133

**Published:** 2020-12-12

**Authors:** Edgar Saavedra, Guillermo del Campo, Asuncion Santamaria

**Affiliations:** CeDInt-UPM, Universidad Politécnica de Madrid, Campus de Montegancedo, Pozuelo de Alarcón, 28223 Madrid, Spain; gcampo@cedint.upm.es (G.d.C.); asun.santamaria@upm.es (A.S.)

**Keywords:** energy harvesting, smart meter, Sigfox, self-powering, autonomous device, energy saving, internet of things

## Abstract

In this work, a novel current metering device was presented. This device was intended to bring current metering capabilities to a wide variety of scenarios: Developing countries, rural areas, or any situation with technological constraints. The device was designed to provide a straightforward installation with no intrusion in the electrical panels. This was achieved by applying energy harvesting techniques and wireless communication technology for data transmission. The device was able to exploit the magnetic field inducted around a wire carrying electricity as energy harvesting, thus acquiring the power it needed to work. Since very low power was harvested, an efficient treatment for the incoming power and a minimal power consumption system were essential. Although exploiting the magnetic fields inducted around a wire has been used for years, the combination of this technology for both energy harvesting and current metering in an end-user device was off-center. To work in a wide variety of scenarios, it used Sigfox for communications as this brought wide coverage and out-of-the-box functioning. The theoretical design of the device was validated by verification assessments for the joint performance of the individual parts compounding the device, including metering capabilities and wireless communication test-bench. Finally, the metering device was tested under three distinct real-world scenarios that demonstrated the viability of the system. Results show that, depending on the metering period and the average current value in the mains line, the device could work forever acquiring and sending electricity consumption data. Perpetual working was achieved with an average current of 3.1 A to meter every 15 min, and an average current of 5 A for a 5-min metering period.

## 1. Introduction

Worldwide electricity consumption is constantly growing: The global increase from 2017 to 2018 was 3.5%, and it has more than doubled from 1990 [1]. Although more trust is put on renewable energies, fuel fossil-dependant energy generation is still a crucial contributor to global pollution, being more than 60% globally in 2018 [2]. Whereas USA and EU have been reducing carbon emissions since the 2000s—both for energy and goods production—emerging economies such as China and India present an increasing CO_2_ contribution trend [3]. To overcome this global issue, there are different strategies such as the use of renewable energies, smart energy consumption analysis to reduce usage, ambient-aware systems, smart grids, or on-demand response policies [4,5,6]. Among them, and focusing on emerging and developing countries, smart metering seems to be the most suitable approach [7]. Apart from being less cost-demanding, it may help to learn about inhabitants’ energy habits, hence possibly reducing consumption and targeting on energy drains [8].

Regarding the smart meters’ market, there are several options working in different ways. Some smart meters are those installed by electric companies to remotely acquire consumption data, and therefore bill accordingly [9,10]. Occasionally, they can even be used as part of the smart grid infrastructure and implement demand response mechanisms [11]. On the other hand, most of the available commercial systems are powered directly by the mains line with a transformer, such as those encountered in [12,13]. Although they have the advantage of also metering voltage (thus, reckoning power factor), this sort of device requires electrical supply, meaning that a specialist is needed for installation and the building power supply might be interrupted temporally, increasing costs and preventing actual deployments [14,15]. Further, in many cases—especially in developing countries or rural areas—electrical board panels face different challenges (location, access, configuration, size, and shape), not allowing the installation of intrusive smart meters. Hence, low cost, easy-to-install solutions may be a crucial factor in the decision for smart meters’ installation. In the literature, there can be found various examples of self-powered electricity meters. Each of them faces a different measure range, electronic configuration, shape, and installation type.

In [16], a stick-on current and temperature sensor was presented, using Zigbee as a wireless technology and for currents in the range of 60–1000 A. Another example can be found in [17], where the authors cleverly implemented a GPS receiver in order to keep time synchronization with the data. Porcarelli et al. implemented an IEEE 802.15.4 compliant smart meter with a back-up battery for consumptions between 10 W and 10 kW, with a maximum measurement error of 1.6% [18]. In [19], a self-powering method based on the vibration of a piezoelectric cantilever excited by the magnetic coupling of AC was presented. A novel development of self-powered microelectromechanical systems (MEMS) current sensor modules was explained in [20], aiming to achieve voltage sensors as well.

However, none of them either used Sigfox as wireless technology or was targeted to ubiquitous, rural, developing areas, as some of them were difficult to install, lacking the aim of being non-intrusive.

This paper is an extension of the work originally presented in the 16th International Conference on Intelligent Environments (IE2020), titled “A Novel, Self-Powered, Non-Intrusive, Sigfox-Enabled Smart Meter for Challenging Scenarios” [21]. In comparison with the IE2020 manuscript, this paper assessed important improvements in the back-end part of the system, changed in the device’s firmware to adapt it to different scenarios, and offered a more detailed analysis and validation of the device. This work presented a novel device that was able to measure current at 230 V single-phase mains lines, with no intrusion in the existing electrical installations, which may be crucial to be deployed in emerging countries or rural areas. Neither wired data connection nor power lines were required to make the system work. All the components of the device, from communications to firmware, were designed to minimize energy consumption and assure perpetual working.

The rest of the paper is organized as follows: Section 2 presents system description, including component selection and design decisions to meet working constraints. In Section 3, the reliability of the device is shown, including results from real-world implementations. Insights coming from results and experimental validation are discussed in Section 4.

## 2. System Design

**Figure 1:**
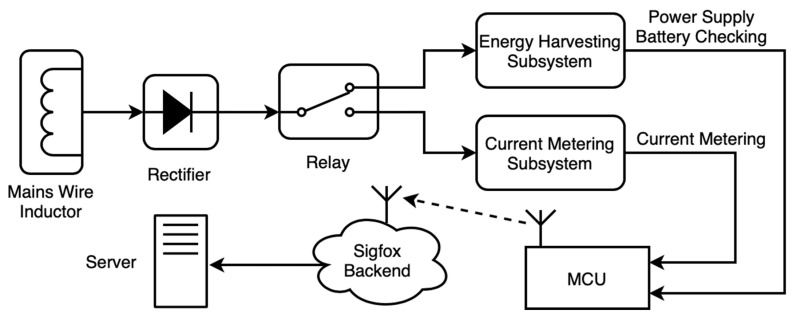
General block-diagram for the complete system, including the metering device itself and the communication to the server.

The aim of the proposed device was measuring currents at mains electrical lines periodically. The general block for the system can be plotted out as in Figure 1. Energy was harvested with a clamp inductor, i.e., a current transformer, which generated an AC current. This current passed through a rectifier, where it was converted to DC before attacking one of the two following circuits: (a) The energy harvesting one, or (b) the current metering one. These two circuits were switched depending on the working state.

If the system was in sleep/stand-by mode, the energy harvesting subsystem was working, supplying power to the microcontroller (MCU) and storing the remaining energy in a battery. Otherwise, the MCU was in charge of reckoning the current value in the mains wire by using the current metering subsystem. Current measurements were stored in the MCU’s RAM (buffer) until a sending event was triggered, when data were parsed and transmitted via Sigfox. The Sigfox backend received these data and then forwarded them to an ad-hoc server, where data were stored and prepared for visualization and analysis.

Sigfox was the selected communication technology, although it presented some drawbacks in terms of packet size (12-byte messages), bandwidth (100 bps), or messages per day (140). Still, it was the most suitable one concerning the smart metering application in different scenarios: Rural environments, crowded buildings, developing countries, etc. Sigfox works out-of-the-box with its own network, providing end-to-end encryption and ultra-narrow band modulation (UNB) [22]. Thus, there was no need to deploy a wireless infrastructure (as with 6LoWPAN or LoRaWAN), which could be ineffective in rural or developing areas with a few smart meters. Cellular technologies such as NB-IoT or LTE-M were also discarded since they were relatively new in the market and they were not really established [23].

The measure range was set to 0–25 A since it seemed to be a fair-minded span for most metering scenarios. Moreover, it allowed each current measurement to be enocoded with just one byte, providing 0.1 A precision—since one byte could represent integers from 0 to 255. This was important to note since Sigfox’s message payload was 12 bytes and measures were sent in groups of 12 to reduce energy consumption. This meant an overall relative encoding precision of 0.4%.

Since the device was meant for a wide variety of non-critical scenarios, such as rural areas or developing countries, where main constraints appear in terms of cost, power consumption, and simplicity, its design was not thought against harsh environments such as industrial facilities or weather hazards. For usage under these conditions, a specific casing should be designed and tested. In any case, using Sigfox assured a high quality of wireless communications, as it was very tough against interferences due to its UNB modulation and receiving antennas’ infrastructure.

### 2.1. Energy Harvesting

Energy harvesting has been a trending topic during the last years, becoming a feasible solution when very-low-power electronic devices were achievable from the 2010s. Providing energy harvesting capability to wireless devices enables them to continuously acquire energy, therefore eliminating the concern of their lifetime being dependant on the energy storage system capacity or battery exchange [24]. Every energy harvesting device is compounded, at least, of three main blocks: The harvester itself, the signal conditioning block, and the storage element.

There are many energy harvesting technologies and new ones may appear in the near future. The most relevant for smart meters and similar applications might be:

- Ambient radiation: This is based on exploiting the large amount of radio frequency (RF) energy available in the ambient at different frequencies; an example can be seen in [25];
- Photovoltaic (PV): Transforming light radiation into current; it is virtually inexhaustible and probably the one from which greatest energy can be obtained [26];
- Piezoelectric: Using the piezoelectric effect, which converts mechanical strain or ambient vibration into electrical energy; an example can be seen in [27];
- Magnetic induction: Electrical energy is obtained by moving magnets—or changing magnetic fields, as in [28]—near or inside a coil;
- Vibration: Vibration energy harvesting may be fitted into the magnetic energy harvesting field, as it usually relies on varying magnetic fields created due to vibrations to generate energy, as designed in [29];
- Pyroelectric and thermoelectric: These are intended to exploit heat in order to obtain electrical energy, whether relying on temperature gradients or time-variant temperatures, as described in [30].

The device proposed in this work was power supplied by means of magnetic induction energy harvesting (MIEH). When magnets teeter through a coil, they create a variable magnetic field. This magnetic field happens to generate an electromotive force (EMF) into the coil. This phenomenon is described in Faraday’s Law of Induction [31,32], which explains the EMF (**E**) created when a time-variant (d*t*) magnetic field (**B**) is present (1, 2). As Faraday’s Law is reciprocal, every conductor carrying a certain amount of current creates a surrounding magnetic field. If this is the magnetic field time-variant, it may be able to be exploited. Thus, some amount of electrical energy might be drawn.

(1) $$\oint_{\partial \Sigma }\;E\;dl=-\frac{d}{dt}\iint_{\Sigma }\;B\;dS$$

(2) $$\nabla \times E=-\frac{\partial B}{\partial t}$$

MIEH seemed to be the best choice for this device considering its own nature, in which a wire carrying electricity had to always be present for metering current. The device needed the existence of a current, and therefore power to perform its current metering function. This device took advantage of the magnetic field happened around a conductor carrying electricity—which was a time-variant as mains electricity was AC—to produce an EMF in a coil. This is the principle used in most current probes for current metering, notwithstanding with the fact that in this work it was also proven to be the power supply for the system. If other methods of energy harvesting were used, a different component would be needed for energy harvesting and metering, increasing the complexity and cost of the device and its functioning. Moreover, depending on the meter location, different types of EH techniques might have been chosen, which counteracted the universal aim of the device.

This approach to MIEH has not been used widely, yet the device proposed in this work and [28] used a similar configuration for the main blocks of the system, but different wireless and switching technologies. It used IEEE 802.15.4 as wireless technology instead of Sigfox, and a MOSFET-based switch system, whilst we relied on a solid-state relay for switching. The device proposed in [28] was proven to self-power itself for a metering period of 60 s when a load of 300 W was connected. In this work, several conditions of the metering period and current consumption were discussed and demonstrated.

### 2.2. Harvester

The harvester itself was a clamp inductor—a current transformer—with a current ratio of 1500:1. This meant that the current outgoing the inductor was 1500 times lower—the secondary coil—than that being carried in the mains wire—the primary coil. The inductor was characterized, including measurements for the short-circuit (SC) current and open-circuit (OC) voltage in the secondary coil. Table 1 shows these measurements, in which the 1500 times relation can be seen—SC current vs. the current through primary. The SC current and OC voltage were the absolute maximum values that the harvester could provide, which were not those really exploited when a load was connected—both of them being lower. In Section 3.1 hereof, actual charging currents are depicted, the top limit being restricted to 1.13 mA.

**Figure 2:**
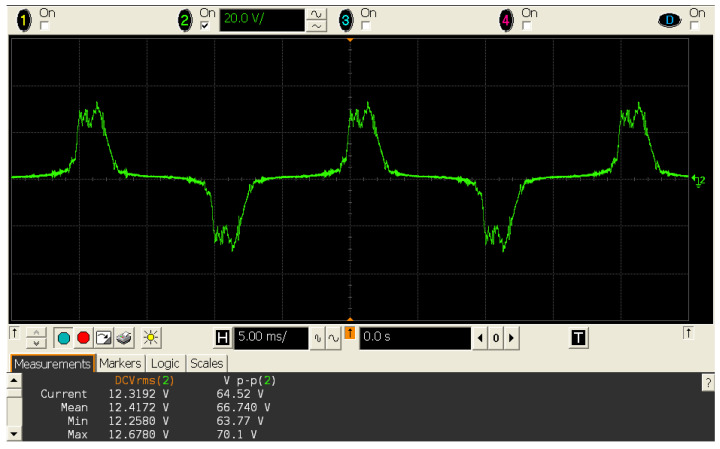
Oscilloscope screenshot where the disturbance occurred due to the inductor’s iron core magnetic hysteresis is depicted.

A worth-noting, non-linear effect occurred in the harvester when it was excited by high currents. The inductor’s iron core magnetic hysteresis altered the sinusoidal shape of the mains wire signal, resulting in a disturbed alternating signal [33]. This effect altered efficiency and dimensioning of the device due to the loss of power capability, thus limiting the harvesting capabilities of the device. Cutting out peak voltage plus sinusoidal shape loss—signal area loss resulted in less usable energy to harvest. It also affected the calibration process since linearity was lost. This anomaly arose when a high intensity magnetic field was inducted into the iron core and it changed, as the material remained magnetized. An example of the signal out of the harvester is shown in Figure 2.

### 2.3. Microcontroller

The MCU had to comply with different requisites that could be summarized in: (i) Very low stand-by power consumption, (ii) Sigfox-compliant, (iii) fast and wide firmware development.

After a market research on Sigfox-compliant development boards, Pycom devices came to court bringing a wide community for development and a MicroPython environment to run the program code. MicroPython is an efficient implementation of Python 3 with a subset of the standard Python library. Further, it is optimized to run on microcontrollers [34]. This leads to a fast, lean, and interactive building process by using Python rather than C, which is the typical language used for microcontroller programming.

Pycom provides several devices depending on the wireless communication. They are all based on the well-known ESP32 [35]. LoPy4 [36] is the one used in this work, which provides Wi-Fi, Bluetooth, LoRa, and Sigfox—although only Sigfox was used so far.

This board provided a deep-sleep consumption of just 25 μA, supporting wake-up from an external interruption and from a timer, which was the one used in this work. Moreover, the integrated 12-bit analog-to-digital converter (ADC) provided 4096 measure points, meaning a theoretical overall precision of 6.1 mA within the 0–25 A measurable range—enough for this purpose and above the 100-mA precision set by the encoding format.

### 2.4. Switching and Conditioning Subsystem

**Figure 3:**
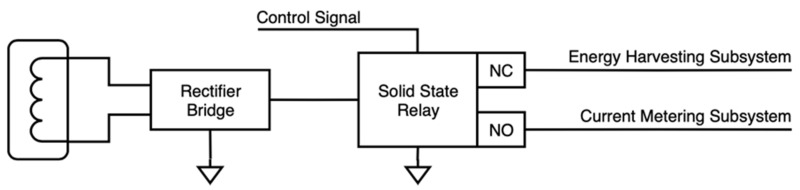
Conceptual schematic of the switching and conditioning circuit, including the harvester, the rectifier, and the solid-state relay.

Since the same clamp was acting as harvester and current probe, it was necessary to have a proper conditioning stage to get the signal ready for either energy harvesting or current measurement (see Figure 3). Considering that this power meter was intended for measuring only active power, and that electronic circuitry worked on DC, the incoming AC signal was rectified from the very beginning to simplify electronic design and procedures. Working with DC allowed us to use a single pole switch—since the other pole is ground—whilst AC would require dual pole switches. Thus, a full-wave rectifier was implemented: CBRHDSH1-40L [37]. Although every component added to the system came with a certain amount of power losses, those resulting from an efficient, full-wave rectifier could be considered negligible.

Once the signal was rectified, it had to go either to the energy harvesting circuit or to the current measurement one. For this switching to be performed, a solid-state relay was chosen: LBA710 [38]. Reduced energy consumption in operation and stand-by was crucial, and a relay guaranteed a non-consuming default state (normally open, NO). Furthermore, using a solid-state relay instead of a magnetoelectric one meant less energy consumption and faster switching.

### 2.5. Energy Harvesting Subsystem

**Figure 4:**
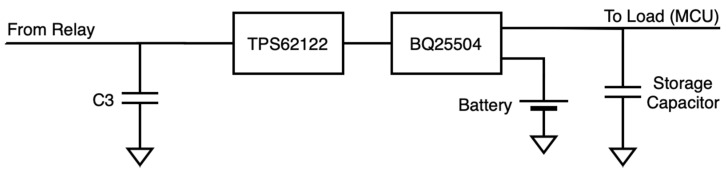
Conceptual schematic of the energy harvesting subsystem.

The harvester subsystem was intended to be as efficient and simple as possible in order to maximize energy harvesting capabilities (see Figure 4). The main element of this block was a power management IC intended for low-power energy harvesting applications: BQ25504 [39]. The BQ25504 is a boost converter that manages the energy storage: The charge of a battery as main storage element and a capacitor as first-stage, instantaneous storage element. The main characteristics of BQ25504 are summarized in Table 2.

The BQ25504 has a restricted input voltage of up to 3 V, but the incoming signal may be higher. Therefore, it is necessary to limit input voltage. Due to efficiency reasons, the buck converter TPS62122 [40] is selected, providing an output of 2.8 V, which is set by means of external resistors. The main characteristics of TPS62122 are summarized in Table 3.

The BQ25504 was configured for an output working range of 3.5–4.2 V, corresponding to the common range of battery (3.4–4.2 V) and MCU (3.5–5.5 V) specifications. These thresholds were set by means of configuration resistors following datasheet instructions.

**Figure 5:**
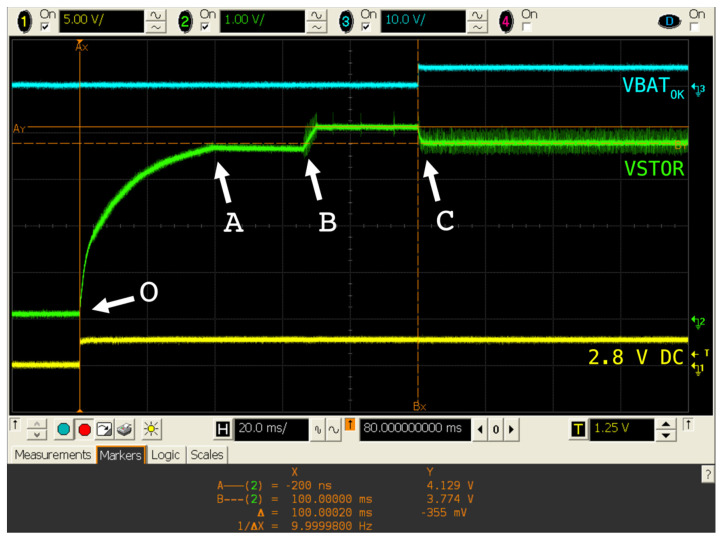
Behavior of BQ25504 when a partially charged battery is attached.

Figure 5 depicts the behavior of BQ25504 when a partially charged battery was attached. The VSTOR signal represents the voltage in the terminal where the first-stage storage element, as well as the load, i.e., the boost converter output, are connected. VBAT_OK_ is a logical signal indicating whether the battery has a reliable level of charge, as determined in the configuration thresholds. The yellow signal was a 2.8 V DC voltage used as the boost converter input.

In instant (**O**), the power source was turned on and therefore the capacitor at VSTOR began charging. The first stage (**O**-**A**) corresponded to the switching of the internal PFET between VSTOR and VBAT, with a duration of ~45 ms. This was the reason why the voltage at point (**A**) was the voltage at the battery, approximately 3.6 V. Then, the charger was disabled for ~32 ms (**A**-**B**), after an internal procedure to reset feedback voltages. Next, VIN_DC_ was used as the power source, being the voltage risen up to nearly 4.2 V (VBAT_OV_) after ~5 ms. Lastly, another ~32 ms later, a normal charging process took place (**C**); thus, the VSTOR voltage dropped to 3.77 V as it was charging the second-stage storage element, and VSTOR and VBAT were short-circuited. The VBAT_OK_ signal switched just when the first feedback sampling was done: Point (**C**), approximately 100 ms after the power source was turned on.

**Figure 6:**
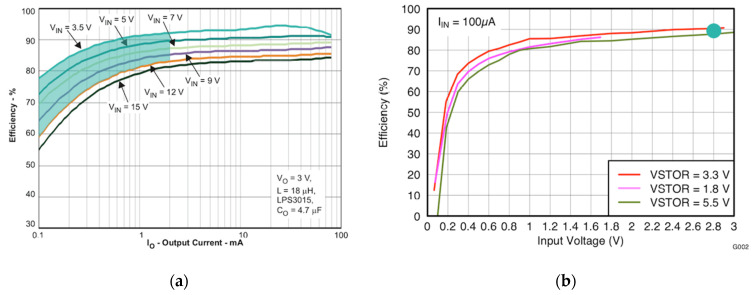
(**a**) Buck converter efficiency curve [40]; (**b**) boost converter efficiency curve [39].

Regarding harvesting efficiency, the two main contributors were buck and boost converters. Figure 6 shows efficiency curves for the buck converter (a) and boost converter (b) for different voltage configurations. In our design, the buck converter was set to provide an output of 2.8 V, which was the input voltage of the boost converter. Output current values were in the range 0.1–1.1 mA, as it will be described in Section 3.1 hereof, and buck input voltages were below 12.4 V, as in Table 1. With these data, energy harvesting theoretical efficiency could be reckoned to be in the range 50–80% (60–90% and 90% for the buck and boost converter, respectively), depending on the working characteristics. However, experimental results showed that real efficiency was lower, resulting in the range of 30–55%.

The power consumption of the energy harvesting subsystem without battery and load was measured and it is depicted in Figure A3 of the Appendix A. It corresponded to an average current consumption of 0.38 µA.

### 2.6. Current Metering Subsystem

**Figure 7:**
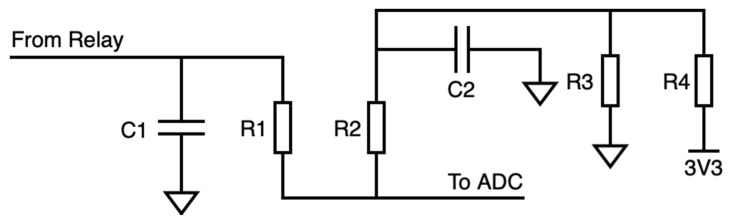
Conceptual schematic for the current metering subsystem.

The current metering subsystem was in charge of measuring the current carried by the mains wire. A measurement resistor was needed for converting the proportional current signal of the probe into a voltage signal for the ADC (MCU) to be read (see Figure 7). Two series resistors were implemented—i.e., a voltage divider (R1-R2)—sampling the voltage in the second resistor of the branch (R2).

The resulting signal was full-wave rectified, yet a capacitor was still to be set in this block. The ripple was desired to be as negligible as possible; but the smaller the ripple was, the longer the stabilization time became. A long stabilization time was not wished as more time would be spent in measuring instead of harvesting; hence, more energy would be wasted. Be that as it may, a perfectly constant signal was not needed as the signal was sampled 1100 times, then averaged, so the ripple was overcome.

**Figure 8:**
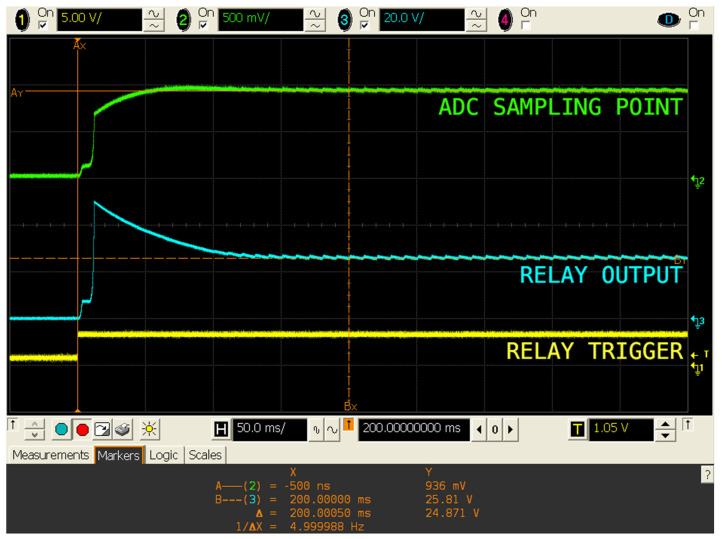
Oscilloscope screenshot: 200 ms convergence time for the metering system.

It is worth noting that, for voltages under 50 mV (0.98 A), the ADC did not work properly, and measurements were very inaccurate, often returning negligible values. To overcome this issue, an offset was set to the signal read by the ADC: A pretend reference was introduced and soared up from ground (GND). This was solved with a voltage divider (R3-R4), using the 3.3 V coming out from LoPy4′s general-purpose input/output (GPIO) pin when the relay was toggled, and stabilized with C2. With a capacitor value of 2.2 μF (C1), a convergence time of 200 ms was obtained, and the ripple reached 15.5 mV in the worst-case scenario, which was that corresponding to the greater current in the mains wire (see Figure 8). The mean value for the ADC signal plus the ripple (green signal, 940.7 mV) remained lower than the ADC limit (1.1 V).

**Figure 9:**
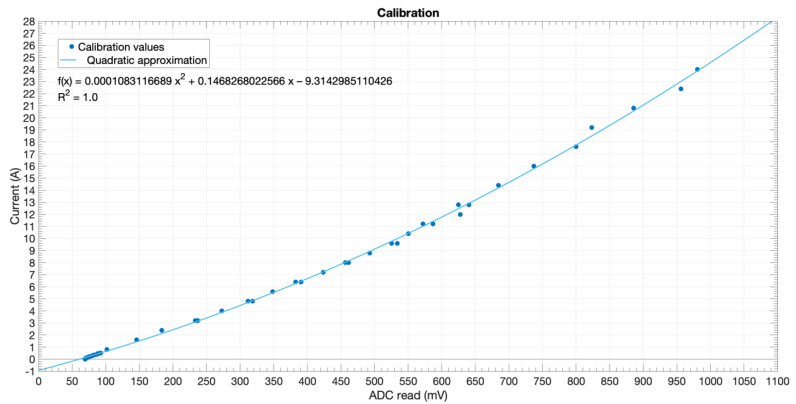
Calibration curve and quadratic approximation.

A calibration process was performed by reading the values returned by the ADC when 48 known currents were sampled (see Figure 9), with an average value of 10,000 samples for each current value. This led to obtaining the quadratic approximation for the current by means of ADC readings with an R^2^ = 1; i.e., a greater order would not necessarily enhance formula’s performance. Note the adjusting of ADC voltages to overcome readings <50 mV: 0 A corresponds to a >50 mV ADC reading. This formula was then programmed in the MCU’s firmware to reckon the current in the mains wire.

### 2.7. Switching between the Energy Harvesting Mode and the Current Metering Mode

**Figure 10:**
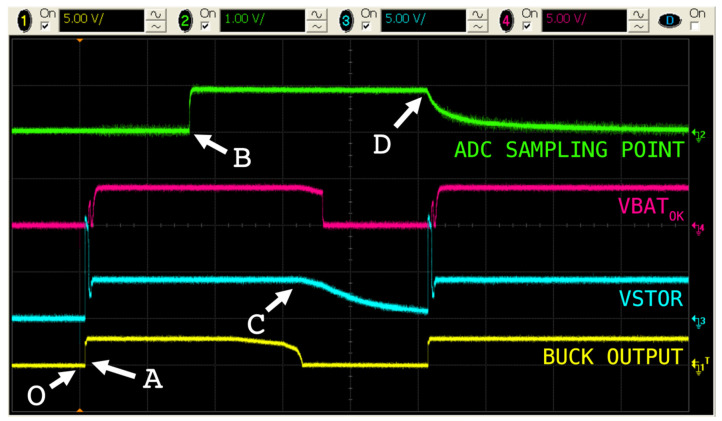
Oscilloscope screenshot: Switching between modes of operation.

As aforementioned, this metering device had two modes of operation: The default one—for energy harvesting—and the periodically triggered mode—to meter the current at the mains wire. The change between states happened when the MCU toggled the solid-state relay. Figure 10 shows the most relevant signals in order to illustrate this phenomenon. Note that there was no battery attached and the process was slowed down in order to see the effect on VBAT_OK_.

In instant (**O**) the load was connected. However, it was not until event (**A**) when there was enough energy in the input capacitor C_DCIN_ so that the buck converter could start working. The switching mode phenomenon occurred at point (**B**), where the relay was toggled. Thus, C_DCIN_ started discharging until occurrence (**C**), where there was not sufficient energy to drive the buck converter and therefore the VSTOR capacitor started discharging. At point (**D**), the relay was toggled, starting the harvesting process anew. As expected, the VBAT_OK_ signal changed its state according to the voltage at the VSTOR and designated thresholds.

### 2.8. Device’s Firmware

**Figure 11:**
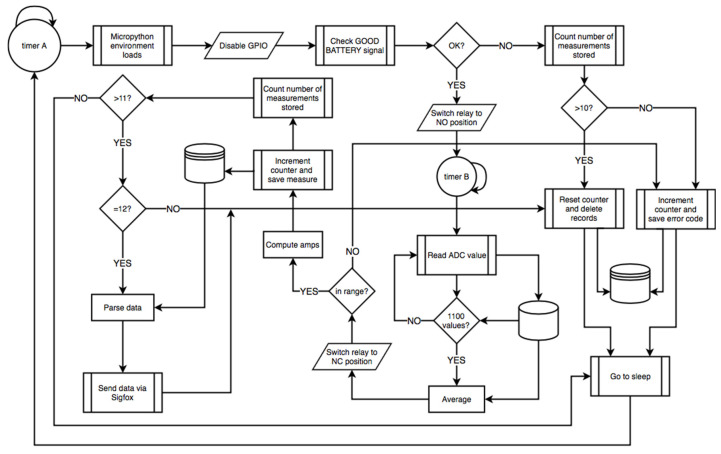
Flowchart for the microcontroller’s (MCU) firmware.

The MCU’s firmware was written in MicroPython, intended to be as straightforward and short as possible to make the processes fast and stable. The behavior was event-driven, based on a 15-min timer to take measurements. Its working principle may be summarized as follows (see Figure 11 for clarification):

1. When the device woke up from sleep mode, it checked the charge level of the battery (VBAT_OK_ signal). If it was above the required threshold, it continued running. Otherwise, error was handled, then it returned to sleep. If this error code provoked the buffer to be full, the buffer would be erased in order not to lose time reference, as it was not sent alongside the message but using Sigfox’s backend timestamp;
2. If the battery health was good, the metering process began. The relay was toggled, then stabilization time was waited. Next, 1100 samples of the signal were collected, averaged, and converted into a current value. If the current reading was out of boundaries, an error message was encoded;
3. The new measurement was buffered and, if the buffer became full (12 measurements), the Sigfox sending process was performed to begin anew.

In order to keep time synchronization, the time the device went to deep sleep and was slightly modified depending on what it did before. Every behavior (metering, metering and sending, low battery level, etc.) took a different amount of time. They all were characterized and taken into account when commanding the device to sleep.

The encoding format, one byte for every measure, is the following:

- [0, 250]: Current reading in tenths of ampere;
- [251, 255]: Reserved for error codes:
  ○ 251: Measure under range;
  ○ 252: Measure over range;
  ○ 253: Future use;
  ○ 254: Handled firmware exception;
  ○ 255: Energy fault, bad battery status.

### 2.9. Server-Side Software

When the device sent a Sigfox message, this was received by the Sigfox backend, where call-backs were set up. In this case, an HTTPS POST request was performed to an ad-hoc server every time a message is received. The usage of HTTPS was crucial in order to send requests encrypted, being only readable by the receiver: A server located at university facilities. The call-back was set to send a header with an authentication token and a JavaScript Object Notation (JSON) body containing the following parameters:

*{“payload”:{“device”:”{device}”,”time”:”{time}”,”data”:”{data}”,”seqNumber”:”{seqNumber}”}}*

The server was written in Node.js [41], using the Express [42] framework on a Raspberry Pi 4 [43]. For communicating with the Sigfox backend, the POST resource was available at https://SERVER-IP:1784/insert. When receiving a request, the server checked the authentication token to prove the request was legitimate. Then it parsed the data, applying the temporal shifting to every measurement and storing them into an InfluxDB [44] database.

**Figure 12:**
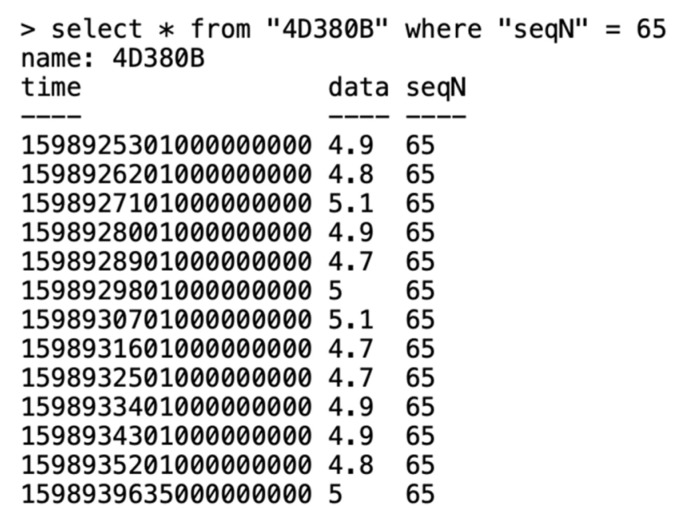
Example of a Sigfox message in the InfluxDB database.

InfluxDB was the selected database technology as it is a very powerful yet fast, emerging temporal-series database. Moreover, it is part of the TICK [45] stack, which provides Chronograf as a straight-forward way of visualizing InfluxDB data, and it is also compatible with the well-known Grafana. In Figure 12, one can see an example of a Sigfox message (12 measures) in the database, where timestamps are stored in nanoseconds referred to as the epoch, data are current measurements in amperes, and seqN is the Sigfox message sequence number.

### 2.10. In-Situ Wireless Communication Latency Test

**Figure 13:**
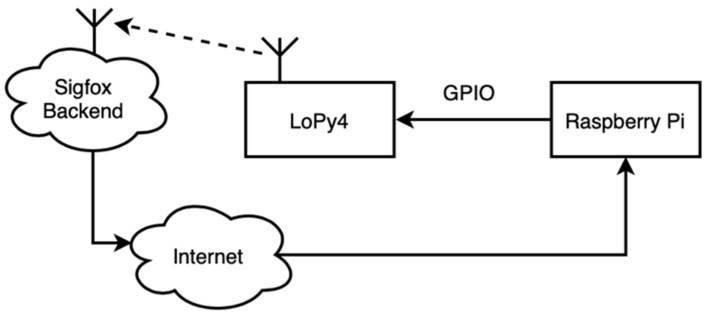
Conceptual diagram of the test-bench deployed for metering Sigfox latency.

We developed a test-bench (see Figure 13) to measure the time elapsed since the device was commanded to send a message until it was received at our server. To do this, we used a Raspberry Pi in which a dedicated Node.js server to measure the latency was deployed. This server rose a GPIO signal to trigger an interruption on the LoPy4 (rising edge). In this moment, the MCU began the Sigfox sending process with a 12-byte payload message and the server timestamps starting time. When the message was received back from the Sigfox backend to the server, the latter measured the time elapsed since the start.

After different tests, the resulting measured latency was about 3.6 s (values between 3.5–3.7 s were measured). From these values, we could extract that a base station was receiving messages in the first Sigfox iteration, since a Sigfox iteration took about 2 s on air for a 12-byte payload message. Sigfox sent messages thrice in three different carriers to ensure delivery. In other geographical zones with worse coverage, this might not be the case, and two or even three iterations could be needed for delivery.

Taking a look into the call-back information, messages were received by four different base stations with identifiers 086A, 080D, 7DC7, and 0663. This meant that the tests were performed in a high coverage area, covered by at least four base stations.

## 3. Results and Validation

In order to verify the proper functioning of the device, three main validation steps were conducted: (i) Power budget validation, (ii) current metering certainty verification, and (iii) real-world application final testing.

### 3.1. Power Budget

The first-proposed metering period is 15 min, thus sending data every 3 h—when 12 measures were collected. In order to guarantee a perpetual working, i.e., no change of battery, for the metering period to be 15 min, a mean current of 3.1 A was needed in the mains line that was being monitored. For shorter metering periods, the average current in the mains line would be greater, as it is explained later.

An Agilent 34,410A multimeter was used to measure and record the device’s working current consumption. There were five main behaviors for the MCU depending on the buffer state and battery level. These were:

1. Sleep mode (15 min, 21.56 μA);
2. Standard measuring process (2.26 s, 59.61 mA);
3. VBAT_OK_ was low, so an error code was saved (1.85 s, 60.55 mA);
4. VBAT_OK_ was low, and this error code saving made the buffer full; thus, it had to be erased and the counter reset (1.81 s, 60.54 mA);
5. A measurement that made the buffer be full was taken, so the Sigfox sending process was launched (12.69 s, 70.57 mA).

**Figure A1:**
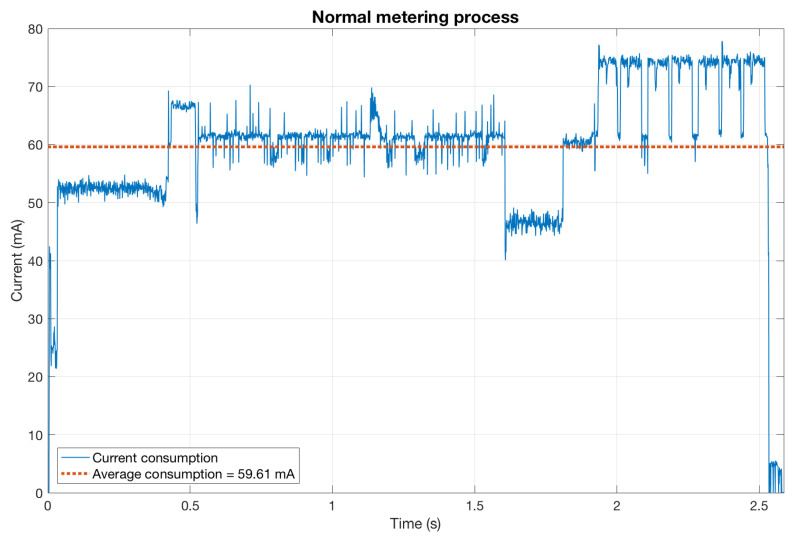
Detailed consumption for the device when a normal metering process occurs.

**Figure A2:**
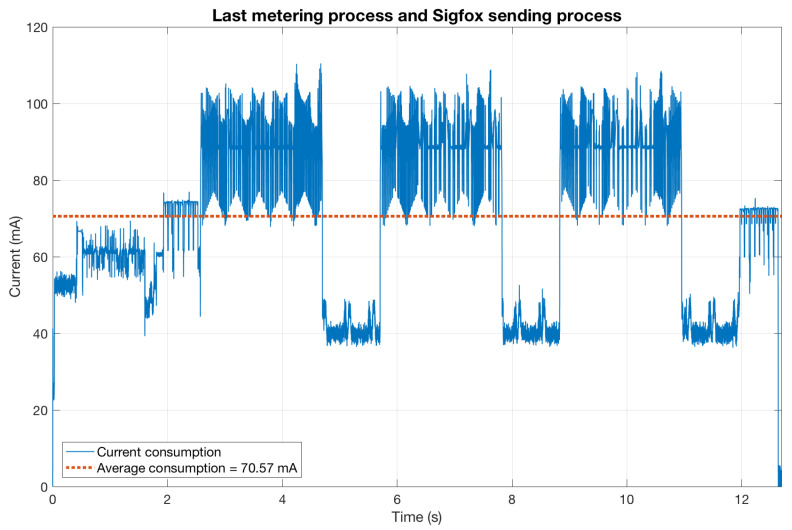
Detailed consumption for the device when a metering process makes the buffer full and the Sigfox message is sent. Notice the three big consumption peaks corresponding to the Sigfox transceiver sending the message thrice—approx. from 2.5 to 12 s.

**Figure A3:**
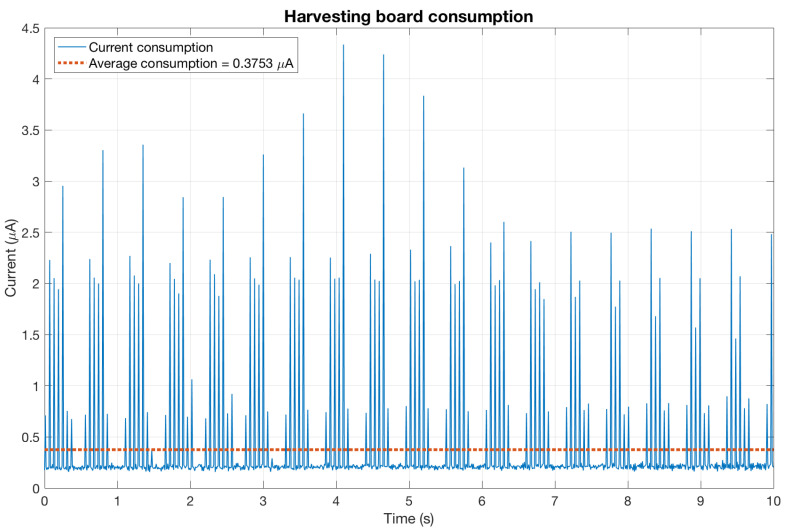
Detailed consumption for the energy harvesting subsystem.

Appendix A presents detailed consumption measurements for processes #2 (see Figure A1) and #5 (see Figure A2), which are the most relevant in energy consuming terms.

**Figure 14:**
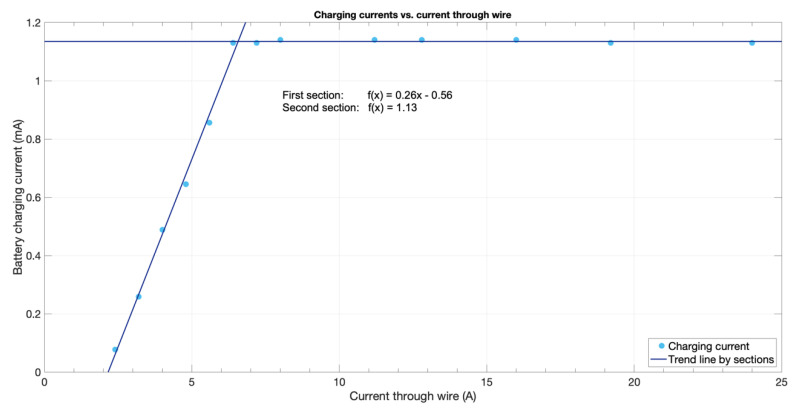
Charging currents for different currents in the mains wire.

Afterwards, the actual energy injected into the battery was characterized, checking the battery life of the device. The energy stored into the battery depended, as previously stated, on the mains wire current. Measured charging currents—for a half-charged battery—are presented in Figure 14.

**Figure A4:**
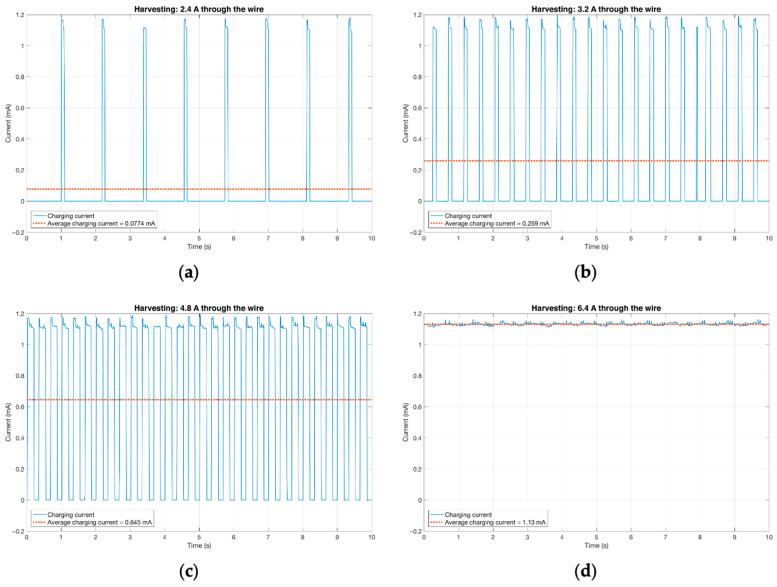
Current injected into the battery: (**a**) 2.4 A in the mains wire; (**b**) 3.2 A in the mains wire; (**c**) 4.8 A in the mains wire; (**d**) 6.4 A in the mains wire.

As it can be observed, the charging current followed a linear pattern until it reached the maximum current that the boost converter was able to inject into the battery. In fact, the charging current behaved as a PWM signal with a top current of approximately 1.13 mA, which increased its duty cycle as the mains wire current increased until 6.4 A (see Figure A4 of the Appendix A for exemplification). This was the reason why once the charging current provided a 100% duty cycle, it could not become higher as the top limit was imposed by the charging circuit itself. Further, for mains wire currents lower than 2.2 A, there was not enough input voltage for the buck converter to start working, therefore stepping down the signal to 2.8 V. This issue might have been solved by using a more complex buck/boost converter or by bypassing the input signal directly to the boost converter if the voltage remained lower than that of the needed threshold.

This issue led to an effective current harvesting range (2.2–6.4 A) different to the whole metering range (0–25 A). This was the reason why, in order to calculate and check power budget, metered currents were assumed to be in the effective current harvesting range. In the upcoming sections, when consumptions were above or below these effective limits, we would be referring to them as the “effective average current” when the average calculation was made, taking into account this limitation—i.e., currents greater than 6.4 A computed as 6.4 A and currents lesser than 2.2 A computed as 0 A. On the other hand, the “average current” referred to the pure mean calculation of the average current.

**Figure 15:**
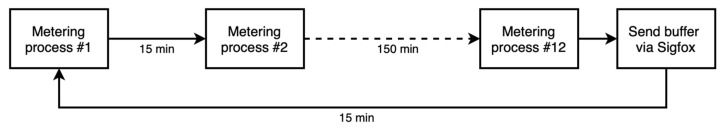
Graphical representation of the standard metering cycle.

With both the consumption and generation characterizations, battery life could now be determined. For this calculation, the most energy-demanding scenario was used, which was also the most feasible one: That where 12 complete measures were done, sending the buffer in the last one via Sigfox (see Figure 15). This energy could be computed following Equation (3):

(3) $$\Psi_{1}=11\cdot 2.26\;s\cdot 59.61\;\mathrm{mA}=1481.9\;\mathrm{mAs}\;\Psi_{2}=1\cdot 12.69\;s\cdot 70.57\;\mathrm{mA}=895.53\;\mathrm{mAs}\;\Psi_{3}=12\cdot 900\;s\cdot 21.56\;\mu A=232.85\;\mathrm{mAs}\;\Psi =\Psi_{1}+\Psi_{2}+\Psi_{3}=2610.28\;\mathrm{mAs}$$

where:

- $\Psi_{1}$ is the energy required for the 11 standard metering processes;
- $\Psi_{2}$ is the energy required for the last metering process which also sends the buffer via Sigfox;
- $\Psi_{3}$ is the energy required for the 12 deep-sleep periods compounding a whole metering cycle.

The aim of this device was being able to work for a lifetime, so the energy harvested into the battery had to compensate for the energy consumed in the process. Taking into account the measured consumptions for every iteration, the necessary current to be injected into the battery happens to be:

(4) $$\Gamma =\frac{\Psi }{t}=\frac{2610.28\;\mathrm{mAs}}{12\cdot 900\;s-\left(11\cdot 2.26\;s+12.69\;s\right)}=242.5\;\mu A\;242.5\;\mu A\underset{harvested\;when}{⇐}\sim 3.08\;A$$

If an effective average current of 3.1 A (713 W, e.g., a workstation) was being carried by the mains line wire, an infinite battery lifetime could be guaranteed for a 15-min measuring period. This average current would increase if shorter periods were wished.

**Figure 16:**
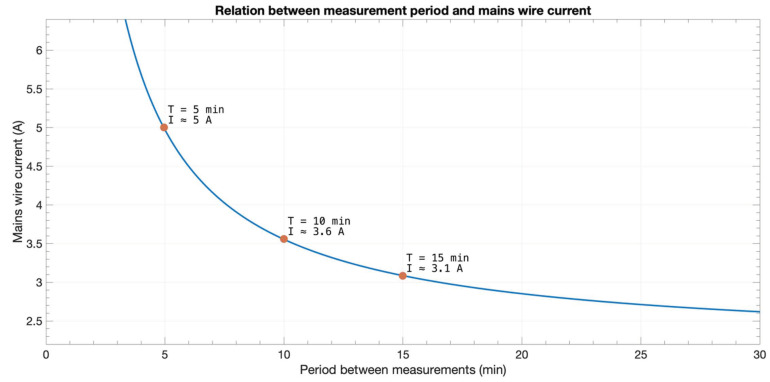
Effective average current needed in the mains wire vs. metering period.

Figure 16 represents the relation between a metering period and efficient average mains wire current. The metering period was a factor from Equation (5)—generalized Equation (4), where T is the metering period in seconds—and the mains wire current came from Equation (6), which is derived from Figure 14.

(5) $$\Gamma \;\left(\mathrm{mA}\right)=\frac{\Psi }{t}=\frac{2610.28\;\mathrm{mAs}}{12\cdot T\;\left(s\right)-\left(11\cdot 2.26\;s+12.69\;s\right)}$$

(6) Γ (mA)=0.26·I (A)−0.56

Working conditions for perpetual work depended on the effective average mains current and the metering period. For instance, for a metering period of 5 min, the effective average current needed would be nearly 5 A. It is worth noting that, due to the 1.13 mA upper limit of the charging circuit, the minimum feasible period was 3.4 min. Further, due to the lower limit from which the circuit began charging (2.2 A), the maximum theoretical period was about 5 h.

**Figure 17:**
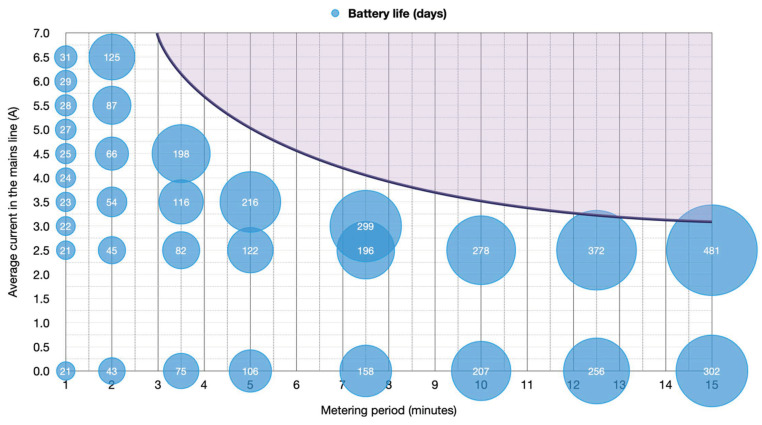
Graphical summary of battery life depending on metering period and effective average current in the mains electrical line.

Considering that the prototype was equipped with a 2200-mAh battery, battery life could be summarized as in Figure 17; where the metering period was depicted on the horizontal axis, average current in the mains line on the vertical axis, and the bubble size showed the estimated battery life for such conditions. As shown in Figure 16, from a certain value and above, battery life would become virtually infinite as the device’s energy consumption became counterbalanced with the energy harvested from the mains (colored area). No bubbles were plotted out for currents less than 2.5 A since the device needed a minimum current of 2.2 A for energy harvesting—as stated in Section 3.1 hereof.

### 3.2. Current Metering

For metering veracity, 15 different current values were probed; composed of 10 consecutive measurements each (see Table 4). Standard deviation was 0.032 A, while the relative error was 3.28% in the whole range of measurement. This relative error was suitable for most cases. Still, it was important to note that it was greatly increased due to the first current values in the lowest range. If measurements below 0.5 A were dismissed, the relative error would result in just about 0.8%. Regarding the standard deviation, it was in fact below the encoding format precision limit (0.1 A), so it was not be noticed. Comparing with the work in [28], they obtained a relative peak error of 1.6%.

### 3.3. Real-World Tests

**Figure 18:**
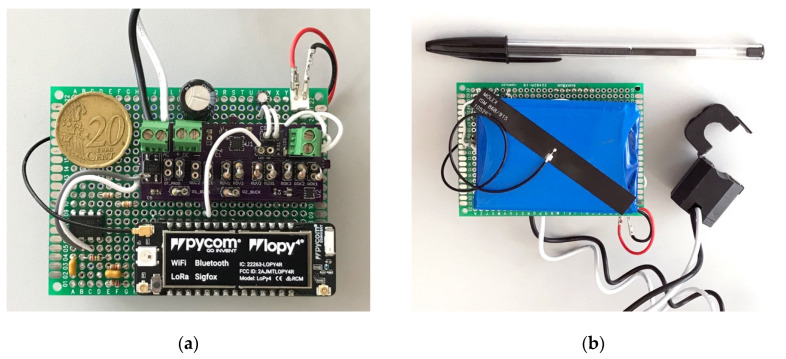
Picture of the prototype: (**a**) Front, where the LoPy4 and the electronic subsystems can be seen; (**b**) back, where the battery, antenna, and clamp inductor are shown.

The prototype (see Figure 18) was put under real-world conditions measuring electrical lines connected to different systems in various environments: A kitchen within the university facilities, an electric water heater, and the main line within a household.

#### 3.3.1. Test #1: 15-Minute Period in a Kitchen

The device monitored an electrical line providing power to a kitchen inside a university building (refrigerator, microwave ovens, water heater, coffee machines, and lighting). During a week period, there were no error messages and the battery level increased from 3.78 to 3.81 V. The effective average current measured during the test was nearly 4 A (920 W). With these energy consumption conditions, the system could work perpetually, and even the metering period could be reduced to around 8 min (see Figure 16).

#### 3.3.2. Test #2: 5-Minute Period for an Electric Water Heater

The device was set to measure the electrical consumption of a water heater. This test was planned because the water heater might be considered as a pure resistive load. Further, its active consumption had to always be the same—which was 1.25 kW according to specifications. The metering period was set to 5 min in order to get more detailed information about the heater’s active timing—having in mind that in this use case, the device would not be able to power itself.

**Figure 19:**
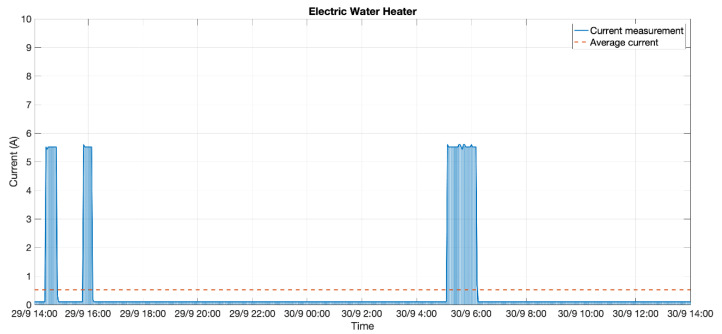
Electrical consumption of the water heater.

In Figure 19, it can be observed that the consumption when the heater was working was similar to that of the specifications (5.5 A, equivalent to 1265 W at 230 V). The stand-by consumption, when resistors were not heating, was 0.1 A. Note that the stand-by consumption could be lower than that as 0.1 A was the metering resolution, as well as the smallest reading value the device could provide.

During the metering period shown in Figure 19 (48 h), the average consumption was around 0.52 A, far from the required ~5 A to ensure perpetual functioning as depicted in Figure 16. In fact, for this consumption behavior, it was not possible to assure a lifetime working condition even though the metering period was enormously stretched.

Taking this 48-h behavior as a permanent, repetitive pattern, we would have:

- 220 min of active consumption (5.5 A), which led to a harvested current of 0.86 mA according to Figure 14, providing 3.15 mAh every 48 h;
- 2660 min of stand-by consumption (0.1 A), which could not provide a harvested current.

The energy required for the device happened to be the same as that of Equation (3), but the stand-by time changed from 900 to 300 s:

(7) $${\Psi^{\prime }}_{1}=11\cdot 2.26\;s\cdot 59.61\;\mathrm{mA}=1481.9\;\mathrm{mAs}\;{\Psi^{\prime }}_{2}=1\cdot 12.69\;s\cdot 70.57\;\mathrm{mA}=895.53\;\mathrm{mAs}\;{\Psi^{\prime }}_{3}=12\cdot 300\;s\cdot 21.56\;\mu A=77.62\;\mathrm{mAs}\;\Psi^{\prime }={\Psi^{\prime }}_{1}+{\Psi^{\prime }}_{2}+{\Psi^{\prime }}_{3}=2455.05\;\mathrm{mAs}$$

Energy computed from Equation (7) was required for each complete cycle, which occurred every hour. Hence, for 48 h, the energy required would be 117,842.40 mAs—32.73 mAh. This energy consumption was countervailed with the 3.15 mAh harvested in the same period, letting 29.58 mAh of net energy consumption every 48 h, which was equivalent to an average current of 0.62 mA.

Therefore, with the 2200-mAh battery used in the prototype—and assuming that a Li-ion battery would provide at least 3.5 V when the discharge capacity was 80%—the device could be autonomously running for 118 days as reckoned in Equation (8):

(8) $$T=0.8\cdot \frac{2200\;\mathrm{mAh}}{0.62\;\mathrm{mA}}=2838.71\;h≅\;118\;\mathrm{days}$$

#### 3.3.3. Test #3: 5-Minute Period in a Household

In order to validate the system in other conditions, the device was put under test in an apartment monitoring its main electrical line. This apartment had a nominal power of 5.75 kW (25 A at 230 V), so it fit perfectly within the measure range of the prototype. After a 7-day electrical consumption dimension test, we assured the mean household consumption was above the designated 3.1 A. In fact, it was around 5.5 A (1265 W).

As the higher average current allowed for shorter metering/sending periods, both the firmware of the device and server parts were modified to collect measurements every 5 min. The device was installed and working as expected for two weeks. During this period, 4000+ current measures were taken and 330+ Sigfox messages sent.

**Figure 20:**
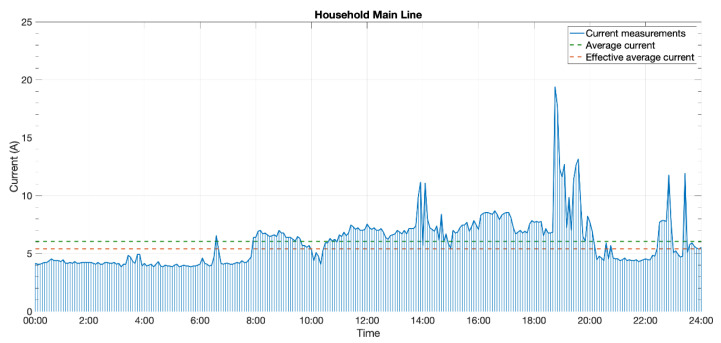
A day of current consumption in the third test scenario.

As an example, a graph corresponding to a 24-h interval was depicted in Figure 20. It could be seen how the 5-min period allowed the detection of electrical consumption peaks, like those appearing around lunch and dinner hours. Even though the average consumption during this period was almost 6.1 A (green line), considering the effective harvesting range, the effective average consumption got to be 5.4 A (orange line)—also satisfying the 5 A lower limit for perpetual working measuring the current every 5 min.

**Figure 21:**
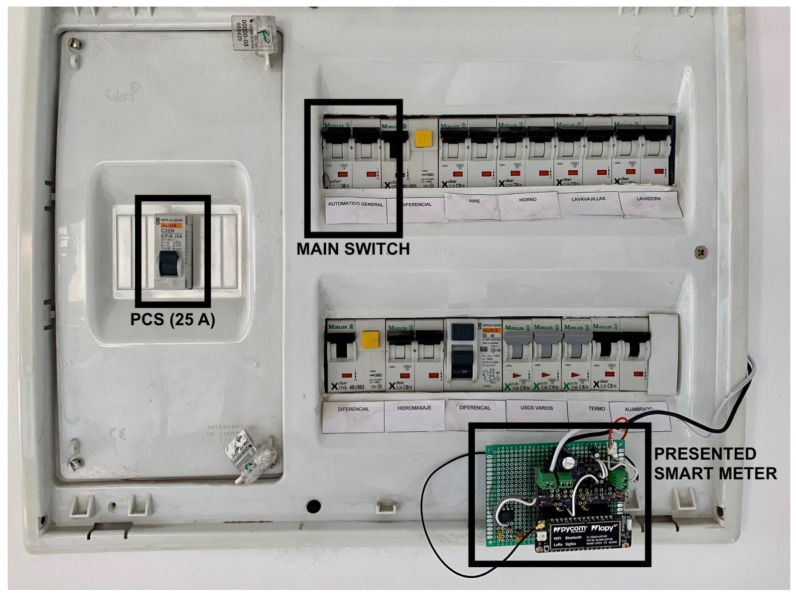
Installation of the device in the electrical panel of a household.

Figure 21 shows the prototype installed at the electrical panel board, where it was clamped to the house main switch. The power control switch (PCS)—left hand side—shows the 5.75 kW nominal power contracted for the house. The installation process did not require powering out, as the only necessary action was adjusting the clamp around the electrical wire.

## 4. Conclusions

Bringing an easy way of metering power consumption was a crucial pillar to counteract the increase of energy consumption and CO_2_ emissions. Having a record on consumptions, people’s consumption patterns, and electricity flows, among other aspects, is essential to detect energy sinks, failures, and irresponsible habits. Such measures are especially relevant in rural areas and developing countries, where access to more advanced strategies, such as smart grids or demand response, are not feasible.

In this paper, a self-powered, Sigfox-compliant smart meter was presented. A fully functional design was demonstrated to work. From the theoretical analysis and real-world tests, we can assure that the device was able to properly meter current and, as long as conditions are complied, work perpetually acquiring current consumption data.

Its electronic design was developed in order to pursue an efficient, simple design, and meet three main requisites: Non-intrusive (easy installation), perpetual working (unending battery lifetime), and global accessibility (scenario versatility and low cost: About 80 € for the prototype, which would be lowered to 20–30 € for a chain-produced device).

However, this device was not a feasible solution for critical scenarios where either very accurate measures or a very short metering period were needed, due to both the energy harvesting technique and Sigfox limitations. Other wireless technologies or power sources would need to be evaluated and tested for such applications. For instance, 6LoWPAN for short distances or LoRaWAN for longer distances could be used if shorter metering periods were wished. Regarding the power supply, PV or vibration energy harvesting could be used together with MIEH if conditions helped it to achieve more usable power.

Regarding metering accuracy, although the overall error rate was about 3.3%, it was lowered to just around 0.8% if the range 0.5–25 A was considered. It is worth noting that, although 0–25 A was the whole metering range, the effective energy harvesting range was 2.2–6.4 A, so the line being metered had to comply with working conditions taking these limits into account, as detailed in Section 3.1 hereof. This range seemed fair for the most common residential scenarios, as it represented a usual current consumption range for a household. However, these limits might be modified, making circuital changes and redesigning the energy harvesting subsystem.

### Future Adaptative Behavior of the Device

Taking into account that the device was intended for a variety of scenarios—average current, consumption peaks, periods with limited current like night-time, energy sampling frequency, etc.—we were interested in applying an adaptive algorithm to the system. The device could be aware of the current measures itself, hence varying the metering period accordingly without the need of a dedicated configuration for every scenario.

For instance, if during a period of time the consumption was almost constant and there was no need for much precision, some current measures could be missed, and the metering period extended. In this manner, energy consumption would be reduced, hence the battery saved. On the other hand, for periods where consumption was higher and variant, more measures could be taken provided that the battery level would recover as well.

Varying the metering period periodically came with strings attached regarding timestamps. With the current firmware, no timestamp was recorded along measurements. A time reference—be it a timestamp, period between measures, or whatsoever—is needed for proper time scheduling if the metering period is wished to be dynamic. This led to not always being able to send 12 measures per Sigfox message, since some payload were needed for time recording.

The device could also be aware of the precise battery level instead of only a VBAT_OK_ threshold. This way, it could be prepared for recovering the battery level when the current is high enough, although it might lose battery charge in other moments when there is less current; however, a short metering period is necessary.

## Figures and Tables

**Table 1:** Measurement samples on the harvester characterization.

$\mathrm{Current}\;\mathrm{through}\;\mathrm{Primary}\;(A_{rms})$	$\mathrm{SC}\;\mathrm{Current}\;(mA_{rms})$	$\mathrm{OC}\;\mathrm{Voltage}\;(V_{rms})$
0.80	0.5	0.7
1.60	1.0	1.5
3.20	2.1	3.7
6.35	4.4	6.8
12.70	8.7	9.5
19.05	13.1	10.8
25.60	17.5	12.4

**Table 2:** Summary of BQ25504 characteristics.

Parameter	Value
Input voltage ($V_{IN}$)	0.12–3 V
Battery voltage (VBAT)	2.5–5.25 V
Storage capacitance ($C_{STOR}$)	4.7 μF
Input power ($P_{IN}$)	0.01–300 mW
Cold-start voltage ($V_{IN\left(CS\right)}$)	300 mV
Min. cold-start input power ($P_{IN\left(CS\right)}$)	15 μW
Switching frequency ($f_{SW}$)	1 MHz

**Table 3:** Summary of TPS62122 characteristics.

Parameter	Value
Input voltage ($V_{IN}$)	2–15 V
Output current capability ($I_{OUT}$)	>75 mA
Output voltage ($V_{OUT}$)	1.2–5.5 V
Switching frequency ($f_{SW}$)	800 kHz

**Table 4:** Measurements corresponding to the metering validation (all values in amperes).

Multim. Reading	1	2	3	4	5	6	7	8	9	10	Avg.	Std. Dev.	Rel. Err. (%)
0.14	0.1	0.1	0.2	0.1	0.1	0.1	0.1	0.1	0.0	0.1	0.10	0.0471	28.57
0.28	0.2	0.3	0.2	0.3	0.3	0.2	0.2	0.2	0.2	0.2	0.23	0.0316	3.57
0.42	0.4	0.4	0.4	0.4	0.3	0.4	0.4	0.4	0.3	0.4	0.38	0.0316	7.14
0.56	0.5	0.6	0.6	0.5	0.6	0.6	0.6	0.6	0.6	0.6	0.58	0.0316	5.36
0.80	0.8	0.7	0.7	0.8	0.8	0.8	0.8	0.8	0.7	0.8	0.77	0.0316	1.25
1.60	1.6	1.5	1.6	1.6	1.6	1.6	1.6	1.6	1.6	1.6	1.59	0.0316	0.63
2.40	2.3	2.4	2.4	2.4	2.4	2.4	2.4	2.4	2.4	2.4	2.39	0.0000	0.00
3.20	3.2	3.2	3.3	3.2	3.3	3.3	3.2	3.2	3.3	3.2	3.24	0.0316	0.31
4.80	4.8	4.8	4.8	4.9	4.8	4.8	4.8	4.8	4.8	4.9	4.82	0.0422	0.42
6.35	6.4	6.5	6.4	6.4	6.5	6.4	6.3	6.4	6.4	6.4	6.41	0.0316	0.63
9.53	9.5	9.6	9.7	9.6	9.5	9.6	9.6	9.5	9.6	9.6	9.58	0.0422	0.52
12.70	12.8	12.8	12.6	12.7	12.7	12.7	12.8	12.8	12.7	12.8	12.74	0.0483	0.24
15.88	15.9	15.9	15.8	15.8	15.8	15.8	15.9	16	15.9	15.9	15.87	0.0316	0.06
19.05	19.0	18.9	18.9	19.0	18.9	19.0	19.1	19.0	19.0	19.1	18.99	0.0422	0.16
23.81	23.9	23.9	23.8	23.9	24.0	23.9	23.9	23.8	23.9	23.9	23.89	0.0316	0.34
**MEAN RESULTS**												**0.0338**	**3.28**
